# Field pea cardinal temperatures, growth and vigor traits of winter- and spring-adapted germplasm at germination and seedling stages

**DOI:** 10.1038/s41598-025-06342-w

**Published:** 2025-07-01

**Authors:** Curtis B. Adams, Juniper Cosner

**Affiliations:** USDA Agricultural Research Service, Columbia Plateau Conservation Research Center, 48037 Tubbs Ranch Road, Adams, OR 97810 USA

**Keywords:** Cardinal temperatures, Field pea, Germination, Pea, Plant growth modeling, Seedling vigor, Natural variation in plants, Plant physiology, Plant ecology

## Abstract

There are few estimates of cardinal temperatures [T_b_ (base), T_o_ (optimal), T_m_ (maximum)] for pea (*Pisum sativum* L.) seed germination and seedling growth traits, with variable results, and there is almost no understanding of how these traits vary among pea varieties or classes. The objective of this controlled-environment study was to compare these among multiple varieties of food-grade winter and spring field peas and Austrian winter pea (AWP). Contrary to hypothesized outcomes, there were no collective differences in any of the three cardinal temperatures between winter- and spring-adapted food-grade field peas. Overall estimates were − 0.98 °C for T_b_, 27.5 °C for T_o_, and 37.5 °C for T_m_. AWP, known for exceptional cold hardiness, contrasted from the food peas in having lower T_o_ (25.7 °C) and T_m_ (36.1 °C). Metrics of seedling vigor and growth (germination rate and percentage, shoot elongation) were markedly higher for AWP than the food peas. The food-grade winter pea seeds were collectively somewhat more vigorous than the spring peas. Considering both cardinal temperatures and germination rate, the average thermal time requirements for 50% germination were 31.0, 46.6, and 55.9 °Cd for AWP, winter food, and spring food peas, respectively. These results provide improved metrics and understanding of field pea seedling responses to temperature.

**Supplementary Information:**

The online version contains supplementary material available at 10.1038/s41598-025-06342-w.

## Introduction

Pea (*Pisum sativum* L.) is a cool-season legume species that has a long history as a cultivated agricultural crop^1^. Phenotypic diversity of the species has been utilized to develop varieties differentiated into several market classes. There are field peas, also called dry peas, with either food- or feed-grade grain market traits; green peas that are consumed as a fresh food vegetable; peas grown as animal fodder as forage and/or hay; and green manure and cover crop peas^2–6^. There seems to be relatively little information about how the different market classes of pea differ physiologically. Furthermore, at least for field peas, germplasm is selected for production in either winter or spring growing seasons, depending on the climate of the intended planting region^7–9^. In the coldest climates, no peas would be expected to survive the winter, thus the plant is grown in the spring in these locations, but sufficient cold hardiness traits exist among pea germplasm for winter survival in more temperate climates. In the relatively severe cold climates of Canada, the Northern Great Plains of the U.S., central and northern Europe, and northern China, for example, spring field peas are typically grown, while in the more temperate climate conditions of the Pacific Northwest of the U.S., southern Europe, and southern China, cultivation of winter field peas is most common^2,9–11^. Notably, Neugschwandtner et al.^12^ and Poudel^10^ described that, due to shifts in worldwide weather patterns, there is increased interest in winter pea production in regions where spring pea production has historically dominated.

With sowing occurring in the fall, winter field peas do not typically experience severe cold at the germination or seedling growth stages. Sufficient cold hardiness in this germplasm is needed to survive the lowest expected growing season temperature, which is usually experienced mid-winter during vegetative growth stages^7,10,11^. Importantly, winter field pea germplasm also needs to avoid frost (i.e., through the timing of phenological development) and/or have some level of frost tolerance in the vulnerable reproductive growth stages to avoid yield loss^8^. In contrast, spring field peas are sown in the late winter or early spring and frequently experience the coldest temperatures of the season at the earliest stages of growth, though generally not as cold as temperatures experienced in mid-winter^13^. Relative to winter field peas, spring peas usually mature in more intense heat- and/or drought-stress conditions, especially in Mediterranean-type climates, and thus have greater need for heat tolerance in later developmental stages^9,11,14^. Peas are generally vulnerable to extreme weather events, such as heatwaves and severe cold, as well as shifts in regional weather, all of which have been documented to occur in-season in pea production regions^15,16^.

To understand and quantify the temperature sensitivities and thresholds of crop germplasm, crop ecologist and physiologists have developed a concept referred to as “cardinal” temperatures^17^. Cardinal temperatures are benchmarks for the base or minimum (T_b_), optimal (T_o_), and maximum (T_m_) temperatures for plant development at any given phenological stage. Estimates of cardinal temperatures are used in multiple types of crop growth models, including complex process-based models and simpler growing degree day models^18–21^. Only a small number of studies have been conducted to estimate cardinal temperatures for pea germination and seedling growth. This includes a study by Bierhuizen and Wagenvoort^22^ that included one variety of green peas (unspecified season adaptation class), a study by Angus et al.^23^ that included three varieties of field peas (unspecified season adaptation class), a study by Olivier and Annandale^24^ on several varieties of winter-adapted green peas, a study by Raveneau et al.^13^ that included several lots of a forage winter pea and two spring peas (unspecified end-use class), a study by Tribouillois et al.^25^ that included two pea varieties (unspecified end-use and season adaptation classifications), and a study by Andrade et al.^26^ that included a single winter pea variety (unspecified end-use class). Table 1 summarizes the cardinal temperature results of these studies. Estimates of average T_b_ varied from − 1.1 to 2.4 °C, T_o_ from 22.6 to 29.5 °C, and T_m_ from 32.8 to 40.4 °C. The variation in these estimates is quite wide, which could be due to differences in pea germplasm physiology, experimental methods, statistical data analysis approaches, experimental error, or other factors. Importantly, many details of the market class and seasonal adaptation of the pea germplasm used in these studies is not specified. There has been little work to directly study whether pea germplasm of different market classes, including end-use and seasonal adaptation classes, differ in cardinal temperatures for germination or differ in seedling growth traits as a function of ambient temperature.

**Table 1:** Cardinal temperature values and ranges (given below averages in parentheses) reported for germination of peas in scientific literature.

Germplasm class	Season adaptation	T_b_^a^	T_o_^a^	T_m_^a^	References^b^
Green (Fresh) peas	Unspecified	2.4			Bierhuizen and Wagenvoort^22^
		(1.6 to 3.2)			
Field pea	Unspecified	1.4			Angus et al.^23^
Green (Fresh) peas	Winter	− 0.40	29.0	40.4	Olivier and Annandale^24^
		(− 1.8 to 0.6)	(28.4 to 29.7)	(40.1 to 41.0)	
Forage/Unspecified	Winter/Spring	− 1.1	22.6	33.1	Raveneau et al.^13^
		(− 2.0 to − 0.5)	(21 to 27)	(29 to 40)	
Unspecified peas	Unspecified	0.35	28.9	32.8	Tribouillois et al.^25^
		(0 to 0.7)	(28.5 to 29.3)	(32.0 to 33.5)	
Unspecified peas	Winter	2.1	29.5	36.2	Andrade et al.^26^
			(27.6 to 31.3)		

^a^Cardinal temperatures: T_b_ = base temperature; T_opt_ = optimal temperature; T_max_ = maximum temperature.

^b^A variety of statistical models were used to determine cardinal temperatures for germination across the referenced studies. Raveneau et al.^13^ and Tribouillois et al.^25^ used models that separately provided estimates of T_b_ and T_min_, though only T_b_ is reported here in the case of Raveneau et al.^13^ and only T_min_ is reported in the case of Tribouillois et al.^25^. Values presented from Bierhuizen and Wagenvoort^22^, Olivier and Annandale^24^, Raveneau et al.^13^, and Tribouillois et al.^25^ represent the average and range of multiple pea variety treatments. Values presented by Andrade et al.^26^ represent the average of the “triangular-shaped” and “plateau-shaped” models for the 0.5 G observations (50% of final germination reached); the range given for T_o_ represents the range determined through just the plateau model.

In summary, few studies have been conducted to estimate cardinal temperatures for pea seed germination, with variable results, and there is very limited understanding of how cardinal temperatures vary across germplasm within the species. Due to differences in the environmental conditions they encounter in the field, we hypothesized that winter- and spring-adapted peas would differ in cardinal temperatures, with winter peas having lower temperature thresholds. Our objective was to test this hypothesis through conduct of a controlled-environment study using winter and spring field peas developed for the U.S. Pacific Northwest region.

## Results

**Fig. 1:**
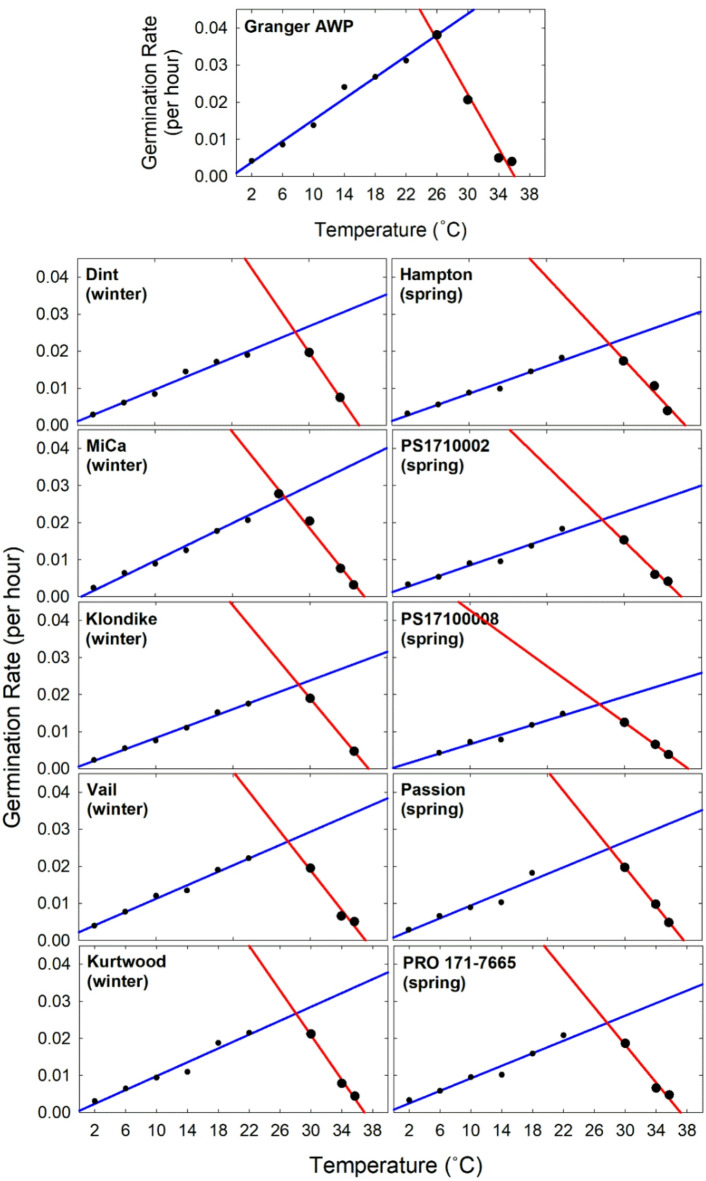
Broken stick plots representing germination rate responses of 11 individual field pea varieties to temperature. These plots were used to estimate cardinal temperatures (T_b_, T_o_, and T_m_) for field peas, as given in Table 2 and Fig. 3.

**Fig. 2:**
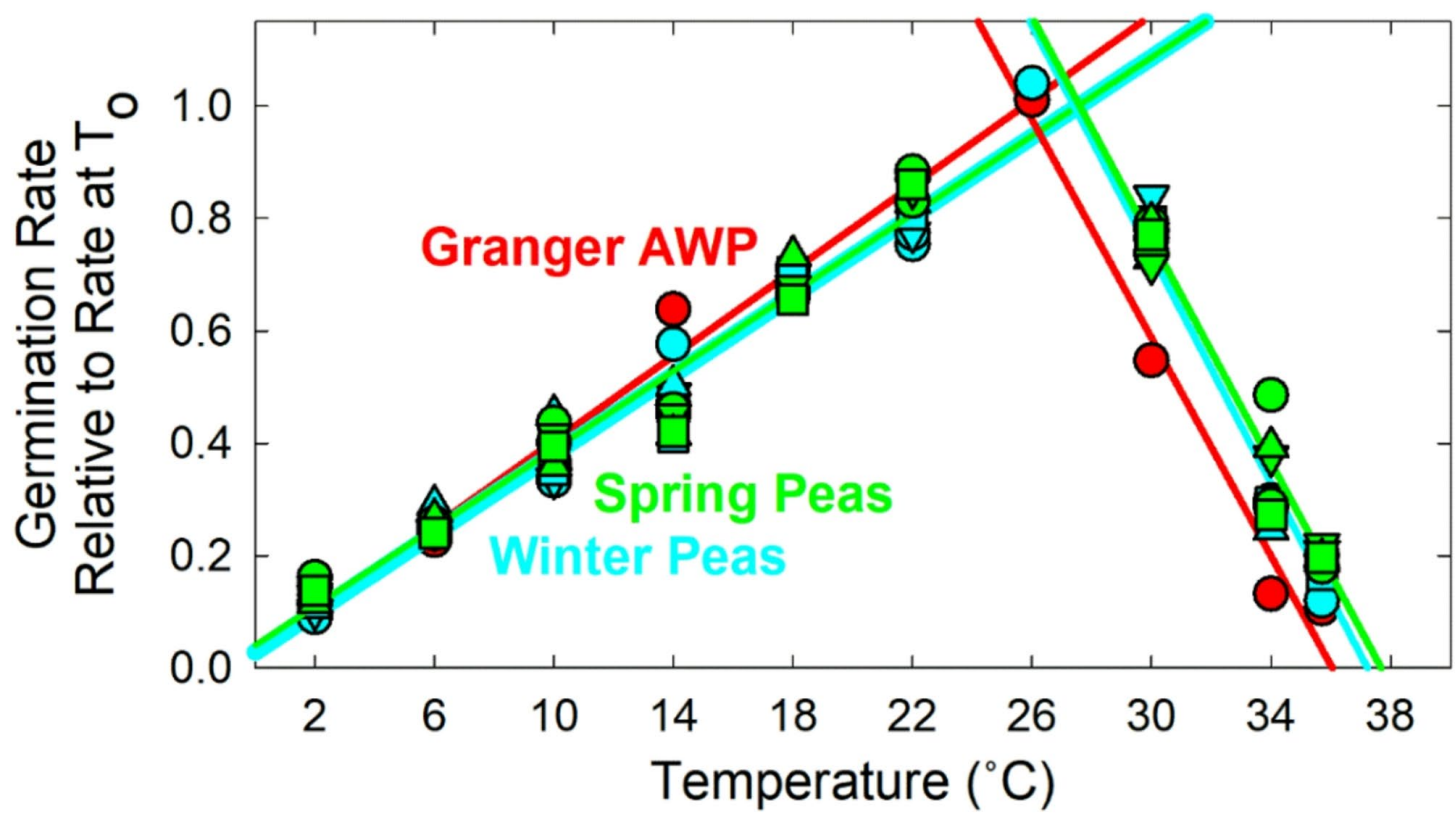
Normalized broken stick plots used to determine and compare collective cardinal temperature (T_b_, T_o_, and T_m_) estimates for field pea classes (winter food peas, spring food peas, and Austrian winter peas (AWP). The cardinal temperature values are given in Table 2 and Fig. 3.

**Fig. 3:**
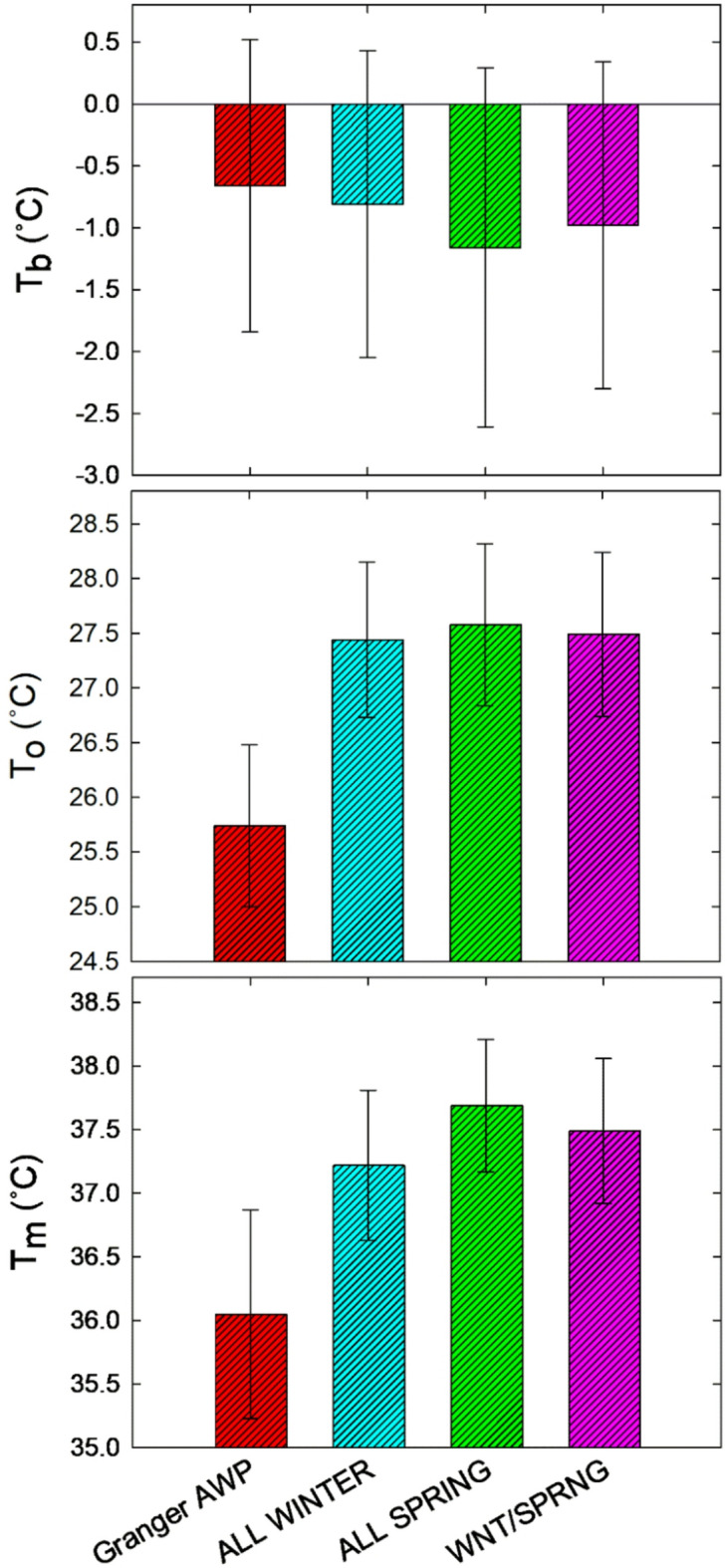
Graphic illustration of field pea cardinal temperatures for germination, including T_b_ (base temperature), T_o_ (optimal temperature), and T_m_ (maximum temperature). This data is shown numerically in Table 2.

**Fig. 4:**
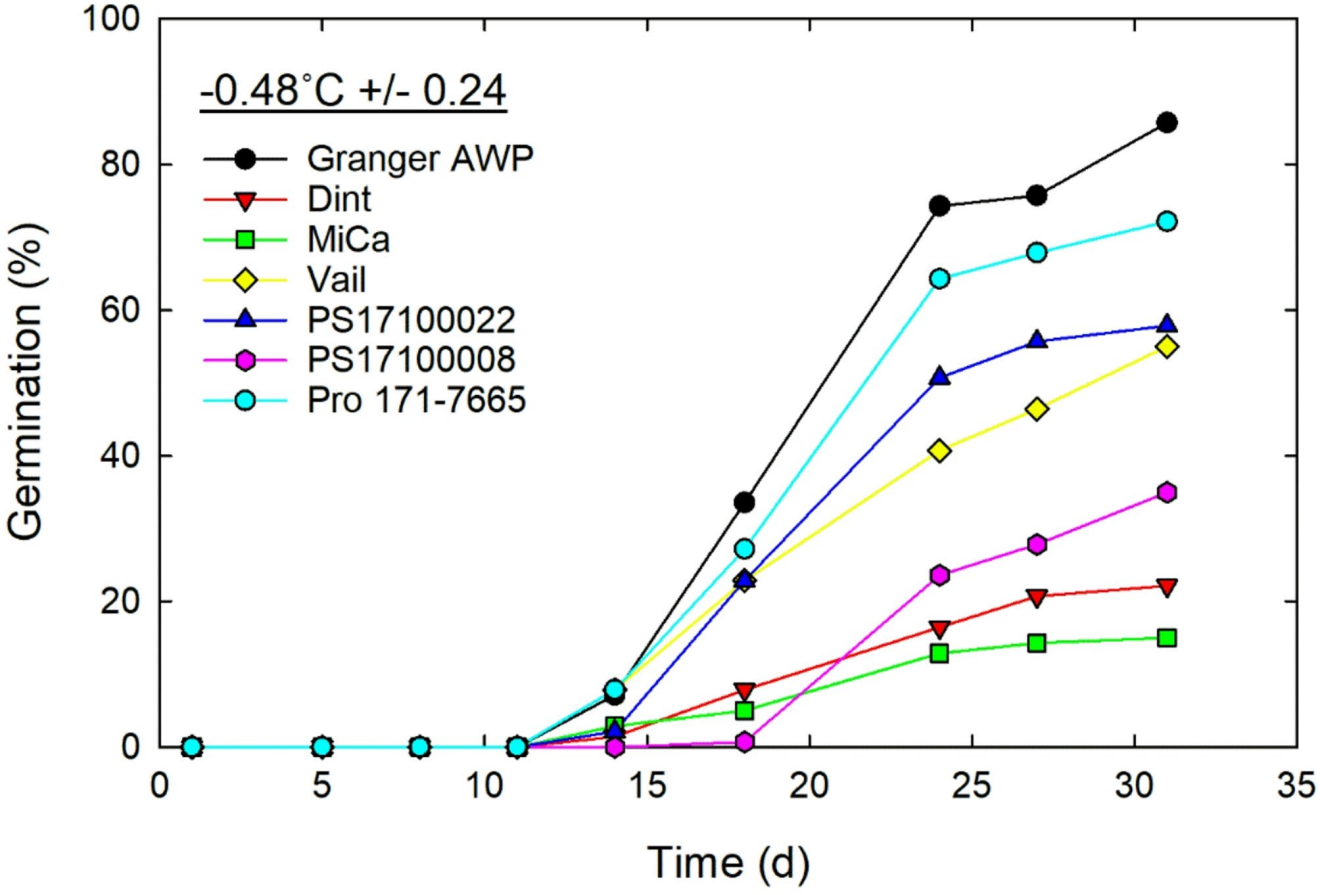
Germination rate data over time for seven select field pea varieties tested at the below-freezing temperature of approximately − 0.5 °C.

Cardinal temperatures (T_b_, T_o_, and T_m_) were determined individually for each tested pea variety and collectively for pea classes, including food-grade winter peas, spring peas, winter/spring, and Granger AWP on its own. Figure 1 graphically illustrates the germination rate by temperature data for each variety, as well as the results of linear regression analyses of the upward and downward trends in germination rate as temperature changed. This figure excludes data points near T_o_ that did not follow the linear trends, thus the full set of data is given in Table S1. Figure 2 illustrates the normalized, collective analyses of pea classes. The cardinal temperature data that was derived from these plots is given in Table 2 and Fig. 3. The T_b_ ranged from − 2.47 to 0.42 °C across all individual varieties, with the winter pea varieties representing both the top and bottom T_b_ estimates. The T_b_ of Granger AWP of − 0.66 °C was among the highest values. When T_b_ was analyzed by seasonal class, there was no indication of class differences. Data from an independent study verified that field peas can germinate at below-freezing temperatures (Fig. 4). Estimates of T_o_ ranged from 25.75 to 28.48 °C across varieties. The variation in T_o_ among winter and spring pea varieties was similar. The T_o_ for Granger AWP (25.75 °C) was considerably lower than all other varieties tested. In assessing differences in T_o_ by seasonal classification, there was no difference between winter and spring peas, though each of these had higher T_o_ than Granger AWP. The estimates of T_m_ ranged from 36.07 to 38.28 °C across all varieties and the variation in T_m_ among varieties within each class was similar. Like T_o_, the T_m_ of Granger AWP (36.07 °C) was lower than nearly all other individual varieties tested. Collectively, T_m_ in winter and spring food-grade peas were not different. Winter food peas did not differ from AWP in T_m_, though spring food peas did, being a significantly higher.

**Table 2:** Cardinal temperatures (T_b_, T_o_, and T_m_) and thermal time (τ) requirements for 50% germination of field peas.

Variety	Adaptation	T_b_^ab^	T_o_^ab^	T_m_^ab^	Τ^cd^
		(°C)	(°C)	(°C)	(°Cd)
Granger AWP^b^	Winter	**− 0.66 ± 1.18**	**25.74 ± 0.74**	**36.05 ± 0.82**	**31.0 ± 5.4**
Dint (ARS)	Winter	− 1.3	28.2	36.5	49.6 ± 3.6
MiCa (ARS)	Winter	0.42	26.9	37.1	39.6 ± 5.4
Klondike (ARS)	Winter	− 0.73	28.5	37.5	53.3 ± 5.9
Vail (ProGene)	Winter	− 2.5	27.1	37.2	46.8 ± 8.2
Kurtwood (ProGene)	Winter	− 0.51	28.0	36.9	43.7 ± 8.6
All winter	Winter	**− 0.81 ± 1.2**	**27.4 ± 0.7**	**37.2 ± 0.6**	**46.6 ± 5.5**
Hampton (ARS)	Spring	− 1.63	28.2	38.0	57.3 ± 6.9
PS17100022 (ARS)	Spring	− 1.77	27.1	37.3	57.3 ± 9.6
PS17100008 (ARS)	Spring	− 0.33	26.8	38.3	66.1 ± 7.9
Passion (ProGene)	Spring	− 0.92	28.0	37.6	49.1 ± 7.8
PRO 171-7665 (ProGene)	Spring	− 0.92	27.7	37.2	49.8 ± 10
All spring	Spring	**− 1.2 ± 1.5**	**27.6 ± 0.7**	**37.7 ± 0.5**	**55.9 ± 7.8**
Winter/sprng	Winter/Spring	**− 0.98 ± 1.3**	**27.5 ± 0.8**	**37.5 ± 0.6**	**51.3 ± 6.3**

Data is presented for 11 field pea varieties, including Granger Austrian winter pea (AWP), five winter-adapted food-grade pea varieties, and five spring-adapted food varieties. Class-specific combined analyses are also given in bold.

^a^Cardinal temperatures (°C): T_b_ = base temperature; T_o_ = optimal temperature; T_m_ = maximum temperature.

^b^The combined cardinal temperature results presented for Granger AWP, ALL WINTER, ALL SPRING, and WNT/SPRNG came from analysis of temperature vs. germination rate data that had been normalized to the peak germination rate calculated at T_o_ for each variety (Fig. 2), enabling valid analysis of cardinal temperatures and associated errors across multiple varieties. The error term is root mean square error (RMSE).

^c^τ: Thermal time (°Cd).

^d^The thermal time estimates, including the class-specific combined estimates, are averages of thermal time required to reach 50% germination across all tested temperatures. The error term is the standard deviation (SD).

The thermal time to reach 50% germination was calculated for each pea variety and averaged within and across seasonal adaptation classes (Table 2). Granger AWP required 31.0 °Cd to reach 50% germination, a rate much faster than all other varieties. The winter food pea varieties ranged from 39.6 to 53.3 °Cd, averaging 46.6 °Cd. The spring food peas ranged from 49.1 to 66.1 °Cd, averaging 55.9 °Cd. The overall average for the food peas was 51.3 °Cd. The error (standard deviation) for individual varieties across tested temperatures ranged from 3.6 to 10 °Cd and averaged 6.3 °Cd overall.

**Fig. 5:**
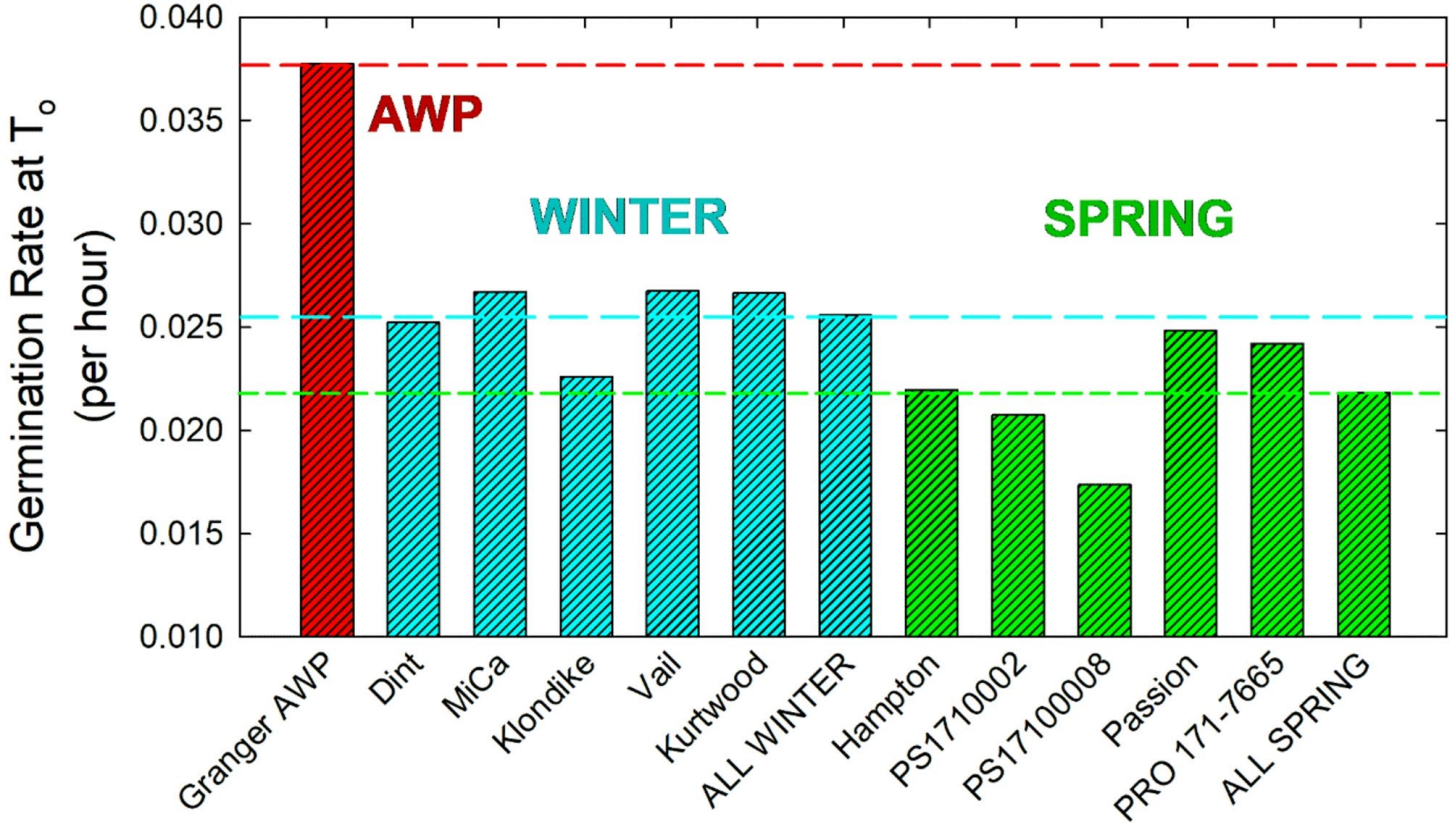
The rate to achieve 50% germination at T_o_, the optimal temperature for germination, as a benchmark indicator of seeding vigor. Data is presented for 11 field pea varieties, including Granger Austrian winter pea (AWP), five winter-adapted food-grade pea varieties, and five spring-adapted food varieties. Averages are also given separately across the winter and spring food varieties (excluding Granger AWP).

**Fig. 6:**
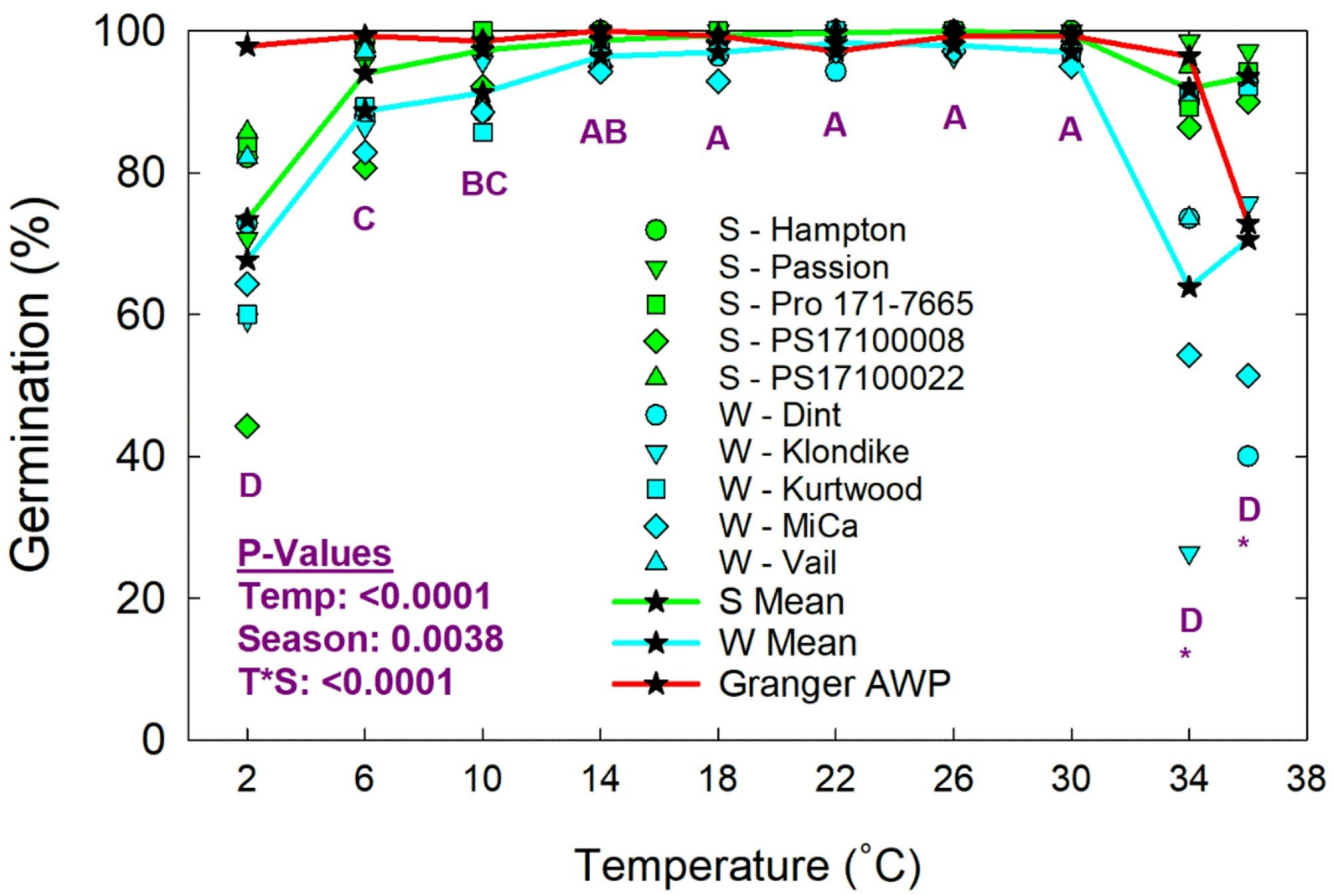
Percent germination of field peas by ambient temperature treatment and seasonal germplasm classification (S = spring; W = winter). The large letters indicate statistical differences among temperatures and an asterisk indicates differences between pea classes (winter and spring) at any given temperature. Results for Granger AWP are presented for comparison but are not included in the statistical analysis.

Data was collected on response variables related to seed vigor, including the maximum rate to 50% germination (Fig. 5) and the percent of seeds germinated by 20 days (Fig. 6). The maximum rate to 50% germination was modeled as the intersection point between upward and downward temperature response linear models at T_o_, as shown in Fig. 1, which will be used here as a benchmark. The peak 50% germination rate of Granger AWP was 0.0378 per hour, which was about 1.6 times faster than the average of all other pea varieties tested. The average peak germination rate of the winter food varieties was 0.0256 per hour. There was a great deal uniformity among winter pea varieties, with only the Klondike variety having a substantially lower peak rate than all others. The spring pea varieties tended to have a slower peak germination rate. For example, their average pea rate of 0.0218 per hour was lower than the rate of all individual winter food varieties, including Klondike. The PS17100008 spring pea variety had a particularly low peak germination rate. The percent of seeds germinated by 20 days varied with temperature, as shown in Fig. 6. Germination was near 100% for all pea varieties from 14 to 30 °C. The percentage of germinated seeds of the food-grade classes declined gradually at temperatures below 14 °C, with a sharper decline at 2 °C. Germination of the winter food pea varieties was significantly negatively affected by higher temperatures, while the spring food pea varieties were not. There were substantial and highly variable declines in percent germination in the winter varieties at 34 and 36 °C. Granger AWP had germination that was near 100% at all tested temperatures, except for a sharp decline at 36 °C that resemble the winter food varieties.

**Fig. 7:**
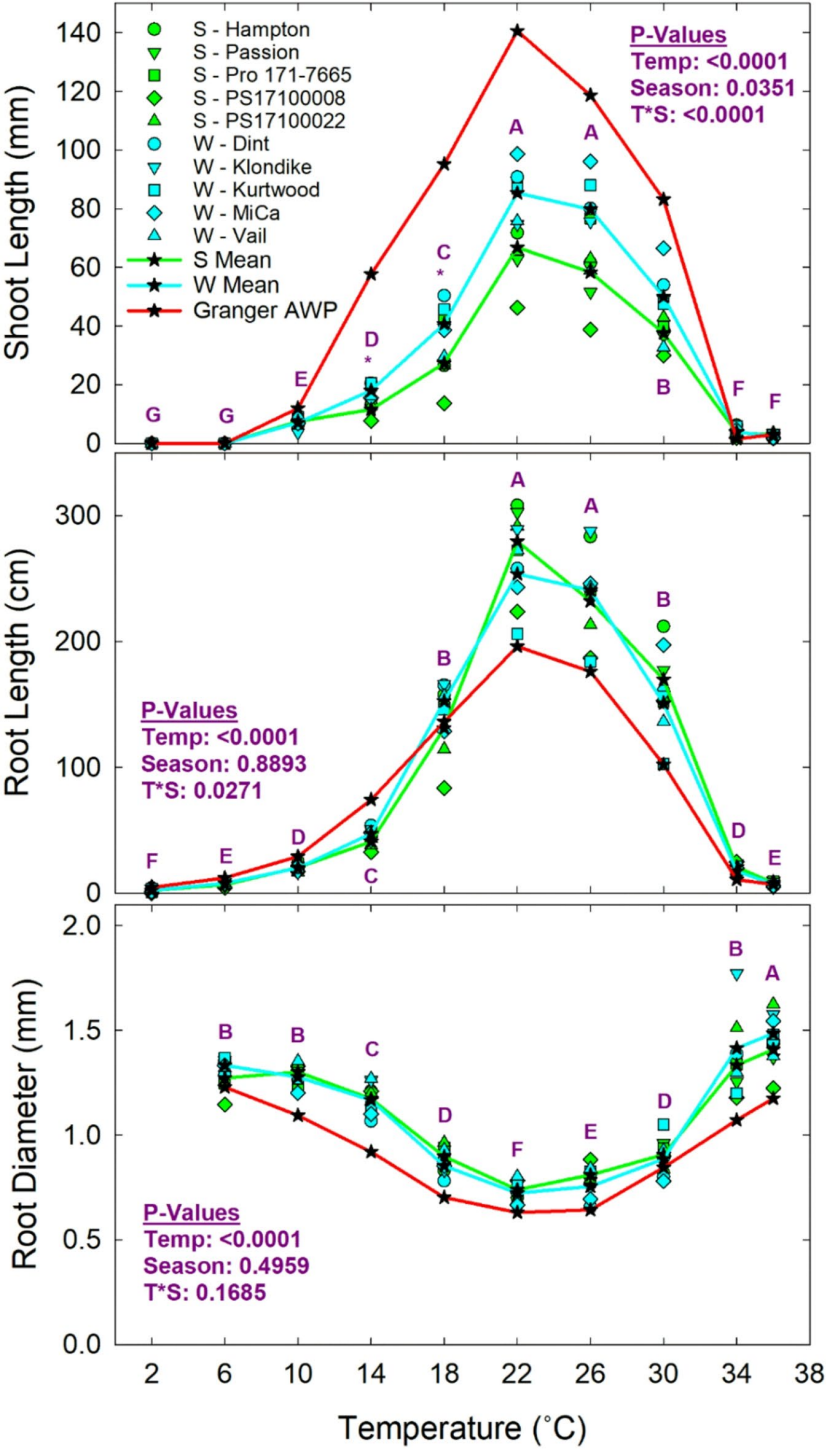
Seedling growth traits of field peas by temperature treatment and seasonal germplasm classification (S = spring; W = winter). The large letters indicate statistical differences among temperatures and an asterisk indicates differences between pea classes at any given temperature. Results for Granger AWP are presented for comparison but are not included in the statistical analysis.

Data on seedling growth traits were collected on germinated seeds at 10 days and statistical comparisons were made by temperature treatments and pea growing season classifications. These parameters included shoot length, root length, and root diameter (Fig. 7). Shoot length was strongly affected by temperature and also affected by seasonal classification. Shoot lengths tended to be near zero at the lowest- (2 and 6 °C) and highest-tested (34 and 36 °C) temperatures. Shoot length peaked at temperatures around 22 and 26 °C. Statistical differences in shoot lengths between winter and spring food pea varieties technically occurred only at 14 and 18 °C, with winter pea varieties having longer shoots. Varietal variability prevented statistical means separation between seasonal classifications at 22, 26, and 30 °C, despite a trend of longer shoots in winter versus spring pea varieties across these temperatures. The shoot length response of Granger AWP to temperature followed the same basic trend as the food peas, but shoot lengths were considerably longer. Like shoot length, root length was strongly affected by temperature with a similar response pattern, but there was no difference between season classifications. There was a relatively sharp peak in root length around 22 and 26 °C. In contrast to shoot length, root length in Granger AWP tended to be lower than the food peas. Average root diameter was also affected by temperature, with relatively coarse roots occurring at lower and higher temperatures, and finer roots occurring at moderate temperatures. There was no impact of seasonal classification. Granger AWP tended to have finer roots than the food peas across all temperatures.

## Discussion

The cardinal temperatures for pea germination had not previously been studied in significant depth. In most prior examinations, test germplasm were often selected without any clear rationale, critical details of the classification (i.e., the end-use and environmental adaptation) of the germplasm were often not specified, and the number of pea varieties evaluated was always very limited^13,22–26^. There have been no targeted comparisons of cardinal temperatures among different classes of pea, either by end-use or seasonal adaptation, to rigorously understand physiological differences and similarities of germplasm within the pea species. As a result, although there is wide variation in published estimates of cardinal temperatures for pea germination (Table 1), there is not a sound basis to identify the reasons for the variation. This makes selection of accurate cardinal temperatures for pea crop growth modeling and other applications challenging. The primary purpose of the current study was to provide a targeted comparison of multiple varieties of modern winter- and spring-adapted food-grade field peas to each other and to AWP. The results shed better light on how environmental adaptation and end-use affect cardinal temperatures and seedling growth responses to temperature in peas.

Within the U.S. market and worldwide, production of food-grade field peas has largely been historically limited to the spring growing season^27,28^. Just in recent years plant breeders in the U.S. started developing food-grade winter field peas to expand production opportunities and potential profitability of the crop^27^. This transitioning germplasm was created by crossing food-grade spring peas with more winter hardy AWPs, then selecting for both food-grade seed traits and plant winter hardiness. The food-grade winter field pea varieties included in the current study are the result of this effort by two separate breeding groups. Before the current study, there were no published results quantifying how the novel germplasm differs in temperature responses or sensitivity at any stage of growth, including germination.

Contrary to hypothesized outcomes, there were no collective differences in any of the three cardinal temperatures (T_b_, T_o_, and T_m_) between winter- and spring-adapted food-grade field peas (Table 2; Fig. 3). There was also overlap between classes in the thermal time required to reach 50% germination, averaging 51.3 °Cd across all food peas (Table 2). These two classes of pea experience drastically different season-long environmental temperature regimes in the field. Spring peas typically experience freezing conditions only at the earliest stages of growth, then may mature under heat stress^9,13,14^. Winter peas are usually planted and establish in relatively warm temperatures, although they can likewise be planted later in colder weather, but they must sustain freezing conditions—often severe cold—through much of the vegetative growth period^29^. Despite the different season-long temperature exposures the pea classes typically experience, the results of this study indicate that this difference does not exert significant influence on their low, optimal, or high temperature thresholds and sensitivities for development at germination. In the process of breeding the food-grade dry peas included in this study, perhaps variation in the weather among breeding field sites and planting dates gave the winter and spring pea selections similar overall exposures to minimum and maximum temperatures during the germination and seedling growth stages, generating similar cardinal temperatures for these stages. There is evidence from various plant species, including peas, that cardinal temperatures vary ontogenically across lifecycles^24,30,31^. Thus, it would perhaps be more likely that cardinal temperatures would differ between winter and spring peas at later stages of growth.

Although none of the cardinal temperatures differed between winter and spring food-grade peas, Granger AWP did differ from the food peas in T_o_ and T_m_ (Table 2; Fig. 3). This gives an indication of the genotypic variation present among diverse pea germplasm in cardinal temperatures, stronger than any variation observed among the individual food pea varieties. The direction of these differences—that AWP had lower T_o_ and T_m_ thresholds than the food peas, especially lower T_m_ compared to spring peas—aligns with the hypothesis that winter-adapted pea germplasm would tend to be more active at lower temperatures and more sensitive to higher temperatures^11,13^. But, interestingly, AWP did not differ from the food-grade peas in T_b_, with each type or class of pea having a similar collective T_b_ that was lower than the freezing temperature. This suggests that a T_b_ of approximately − 1.5 °C may be a lower-limit for the species, especially since there are few reports of T_b_ values lower than this (Table 1)^13,24^. The data shown in Fig. 4 directly demonstrates that both spring- and winter-adapted field peas can germinate at temperatures below the freezing point. Several previous studies have generated estimates of pea T_b_ greater than 2.0 °C^22,26^, which may represent less cold-adapted germplasm, artifacts of experimental approaches, or other error. As detailed before, previously reported average estimates of cardinal temperatures for pea germination have quite wide ranges: − 1.1 to 2.4 °C for T_b_, 22.6 to 29.5 °C for T_o_, and 32.8 to 40.4 °C for T_m_ (Table 1). These ranges partially represent actual variation in pea germplasm traits, but it also seems likely that differences in analytical approaches, limited experimental scopes, and experimental error across studies are also significant contributors.

In addition to generating estimates of cardinal temperatures, data was also collected on seedling vigor and growth traits. A surprising finding of this study was that Granger AWP had a strikingly higher rate of germination compared to all winter and spring food-grade field peas (Fig. 5). This translated into a lower thermal time requirement for germination (Table 2). Granger AWP also had the highest overall germination percentage (Fig. 6). Given that food-grade field peas overall had reduced seedling vigor compared to AWP, reduced vigor may be associated with the food-quality character of the seeds. There is at least some evidence that pea seedling vigor can vary among germplasm differing in seed color or other traits^32^. A fast germination rate and high germination percentage are both traits that lead to successful crop stand establishment and the associated agronomic benefits^33^. In a soon-to-be published report, Adams^34^ tested Granger AWP, Klondike, and MiCa field pea varieties in a multi-environment field trial and collected season-long data on plant stand density, grain yield, and other parameters. In that study, stand establishment of Klondike was relatively slow and poor in a season in which cold conditions came shortly after planting, which ultimately negatively affected yield. The results of the current study indicate that the T_b_ for Klondike is similar to Granger AWP and actually lower than MiCa (Table 2), but the germination rate (or vigor) of Klondike was lower than MiCa and, of course, much lower than AWP (Fig. 5). It seems that lower seedling vigor, rather than T_b_ (or cold sensitivity) per se, may have been responsible for the establishment challenges of Klondike. In comparing winter and spring peas collectively in seedling vigor traits, winter peas seemed to have a slight advantage in germinate rate, while spring peas had an advantage in germination percentage, though just at high temperatures. These trends make sense. Vigorous germination would be beneficial to winter pea to enable stand establishment before long-lasting harsh winter conditions set in. Spring peas may be better adapted to hot conditions overall, since they get more exposure to heat.

There were also important differences among the pea germplasm in other seedling growth traits (Fig. 7). The shoot length (or rate of shoot extension) was greater in food-grade winter peas than spring peas across favorable growing temperatures. Rapid shoot elongation is expected to disproportionately benefit winter peas over spring peas, again, as they push through the soil and establish a strong shoot before winter conditions set it^29,35,36^. Granger AWP had much longer shoot length than even the food-grade winter peas. The AWP class of field pea germplasm is known for robust winter hardiness, which has been associated with early development of a rosette of tillers^27,36^. Although AWP germplasm is a parent of the novel food-grade winter field pea varieties^27^, this data indicates that much of the vigorous shoot elongation character of the AWP germplasm was not retained. Interestingly, there were no differences between winter and spring food peas in root length, although AWP tended to have lower root length than these. Perhaps with the strong early investment of AWP in shoot growth, root growth is diminished. It is notable that AWP also had finer roots (narrow diameter) than the food peas. Finer roots extract water from a larger volume of soil relative to their mass than coarse roots^37^, thus another biological tradeoff among varieties may be apparent in this data. The root diameter data showed the negative impact that both low and high temperatures had on root elongation across all types of peas, which would hamper plant growth and fitness.

As noted in the Introduction, Neugschwandtner et al.^12^ and Poudel^10^ described that winter pea production is of interest or already increasing in regions where spring pea production has historically been dominant. This trend is being driven by shifts in global weather patterns. Many regions are experiencing warming trends, as well as increased frequency of extreme temperature events, both hot and cold^15,16^. Management adaptation, such as shifting production from spring to winter, may be necessary to bolster crop yields in areas experiencing warming and/or drought. Understanding and utilizing genetic variation among pea germplasm in temperature tolerance and sensitivity is also critical. The data collected in the current study illustrates that any increases in temperature above T_o_, averaging a relatively low 25.7 °C for AWP and 27.5 °C for the spring and winter food-grade peas, will have negative impacts on pea seed germination and seedling development (Table 2). Temperatures of approximately 34 °C or higher will have severe negative effects. At these high temperatures, germination percentage declines (Fig. 6), seedling and shoot growth come to a halt (Fig. 7), and seed and seedling mortality increase markedly (data not shown). This was true of both winter- and spring-adapted peas, though there was indication that T_m_ was greater for some spring-adapted pea varieties (Table 2). Should extreme heatwave events occur, such as the heatwave that affected the Pacific Northwest of North America in 2021^16^, negative agronomic consequences on peas are likely unavoidable.

## Methods and materials

### Experimental design and procedures

This experiment was conducted at the USDA Agricultural Research Service (ARS) Columbia Plateau Conservation Research Center (CPCRC) near Pendleton, OR in 2024 and 2025. Two incubators (SG30 Controlled Environment Chamber, Hoffman Manufacturing, Corvallis, OR), equipped with both chillers and heaters, were used to implement the study. Experimental treatments included steady-state ambient temperatures (10) and field pea varieties (11). The temperature treatments were: 2, 6, 10, 14, 18, 22, 26, 30, 34, 36 °C. The field pea variety treatments included an Austrian winter pea variety (1), plus field pea varieties considered food-quality by U.S. standards that were bred as winter- (5) and spring-grown (5) types. The individual pea variety names and their sources are given in Table 2. The experimental design was a randomized split-plot design, with temperature as the main plot factor and pea variety as the sub-plot factor. Each temperature treatment was run independently in a single incubator, with all pea variety treatments included. The experimental units were germination boxes that each contained 35 seeds of a single pea variety. There were four replicate germination boxes per pea variety per temperature treatment. Thus, there were 48 experimental units per temperature treatment, collectively including 140 seeds of each pea variety. The placement of germination boxes were completely randomized within the incubator and were rerandomized daily as germination checks were made, as described below.

Incubator temperatures were independently verified and monitored using 109 thermistor-based temperature probes (Campbell Scientific, Inc., Logan, UT, USA). The temperature probes were connected to a CR1000X datalogger for sensor control and data storage, and the PC400 datalogger support software was used in conjunction for data monitoring and download (Campbell Scientific, Inc., Logan, UT, USA). The temperature probes were attached to the bottom of a central shelf within each incubator to represent average conditions. Prior to beginning any run of the study, the incubator was set to the desired treatment temperature, then adjustments to the setpoint were made using data from the external temperature probe to closely achieve the desired temperature. Data was logged at an hourly interval over the entire course of each temperature iteration of the study. The hourly average, minimum, and maximum temperatures recorded across each temperature iteration of the study are given in Table 3.

**Table 3:** Measured incubator temperatures at 10 treatment set points, including the average (± standard deviation), minimum, and maximum over the period of each temperature iteration of the study.

Treatment (°C)	Avg. Temp.^a^ (°C)	Min. Temp (°C)	Max. Temp (°C)
2	2.07 ± 0.22	1.81	3.96
6	6.08 ± 0.27	5.77	7.98
10	10.09 ± 0.15	9.77	10.95
14	13.99 ± 0.13	13.73	14.54
18	18.03 ± 0.08	17.75	18.16
22	22.07 ± 0.07	21.73	22.24
26	25.92 ± 0.06	25.77	26.08
30	30.12 ± 0.25	28.48	30.36
34	34.01 ± 0.34	31.27	34.29
36	35.65 ± 0.38	32.10	36.39

^a^*Temp.* temperature; *Avg.* average; *Min.* minimum; *Max.* maximum.

The germination boxes were transparent acrylic with a snug-fitting lid and had dimensions of 241 × 160 × 38 mm (l*w*h) (Hoffman Manufacturing, Corvallis, OR). The boxes were prepared for each run of the study (temperature treatment) by thoroughly washing and sanitizing. The pea seeds were stored at 4 °C. At least three days before they were placed inside germination boxes for testing, the seeds needed for a study iteration were pre-counted and then placed dry inside an incubator set to the test temperature. This was done to allow advanced adaptation of the seeds to the temperature treatment. When it was time to begin a study iteration, two sheets of blue germination (blotter) paper (Hoffman Manufacturing, Corvallis, OR), sized to fit the germination boxes, were placed inside. The top sheet of germination paper was modified by drilling 35 holes of 6.4 mm diameter, laid out in a grid pattern of five seeds by seven seeds, with 32 mm distance between hole centers horizontally and 25 mm vertically. The paper was thoroughly wetted with tap water, then extra water was decanted off. Seeds were surface-sterilized by placing them in a tap water solution of 20% bleach and 0.05% Triton X-100 surfactant (EMD Millipore Corp., Burlington, MA) by volume for 15 min. After bleach treatment, seeds were actively rinsed three times, using separate 0.5 L volumes of tap water to remove the bleach. The sanitized seeds were then placed within the wells created by the holes drilled in the top sheet of germination paper. A treatment-identifying label was placed on the lid of each germination box in the upper lefthand corner. Individual seeds within the boxes were identified by their column and row placement, using A through G for the seven columns, and 1 through 5 for the rows, with seed A1 located in the corner by the label.

The moisture level in the boxes was monitored and controlled gravimetrically throughout the study runs. A preliminary test was conducted to determine a gravimetric moisture set point for the germination boxes. This was done by preparing 12 germination boxes as just described. These were assigned to contain seeds from four randomly selected pea varieties, including three replicates of each. The seeds were subjected to the surface sterilization procedure before placing them in the boxes so their moisture status would reflect actual study conditions. Once prepared, the germination boxes and sterilized seeds were weighed without their lids. Analysis of the weight data indicated there was a small standard deviation across all boxes (± 3 g), thus a single weight set point was determined that was applied uniformly for all pea varieties. The box weight or water level was always checked one day after initiating a study run by weighing the boxes on a balance. A squirt bottle was used to add water, as needed each time the germination paper visually showed any sign of dryness, to bring the water level to the set point throughout study runs.

The pea seeds were monitored daily for germination using the letter-number seed identifiers for each box. This was done until germination was complete, up to 20 days. A seed was considered germinated when the radical emerged from the seed coat was at least 1 mm in length^13^. Seeds that showed clear signs that they were dead (e.g., microbial growth), were removed from the boxes and noted as dead. Ten days after starting a study run, six germinated seeds were removed from each box so root and shoot growth and morphological parameters could be analyzed. Seedlings were selected to be representative of the range of growth states found within each box. If fewer than six seed has germinated, a smaller number was analyzed. Please note that this analysis represents the growth status of only germinated seeds at 10 days and does not represent overall seed conditions, as there were often ungerminated seeds at this point. The six roots were cut at the seed surface, then analyzed collectively using scans and analysis by the WinRHIZO Pro software (Regent Instruments, Quebec City, Quebec, Canada). Measured parameters included total root length and average root diameter. The shoot length of the six seedlings were individually measured using digital calipers. If all living seeds had germinated within 10 days, the study run was complete. If not, the run continued until all living seeds had germinated, up to 20 days.

Select pea varieties (Granger AWP, Dint, MiCa, Vail, PS17100022, PS17100008, Pro 171-7665) were also tested for germination below freezing, at approximately − 0.5 °C. The same experimental procedures were used as described above, except no root or shoot data was collected and the interval between germination observations was less frequent. Observations were made over a longer one-month timeframe in this case. This data was used to test the prediction that germination could occur below freezing and was not used in any modeling of cardinal temperatures.

### Data and statistical analysis

Data and statistical analyses were implemented using the SAS 9.4 software package (SAS Institute Inc, Cary, NC, USA), the SigmaPlot 15.0 software (Systat, Inpixon, Palto Alto, CA, USA), and Microsoft Excel (Microsoft Corporation, Redmond, WA, USA).

The germination data for each pea variety by temperature treatment was used to determine cumulative percent germination over time. The Gompertz function was used to model cumulative percent germination as a function of time^13,38^, pooling all replicates, using SigmaPlot (Sigmoidal, Gompertz, 3 Parameter) as follows:

$$G\left( x \right) = a*{\text{exp}}\left( { - \exp \left( { - \frac{x - x0}{b}} \right)} \right)$$

where $G\left( x \right)$ is the cumulative germination percentage at time $x$ from the start of the germination test in hours, $a$ is the asymptote or theoretical maximum for $G$, $x0$ is the time of maximum germination rate or the time of curve inflection, and $b$ is a shape parameter. The results of the analysis were used to calculate the time at which 50% germination occurred. The time that was determined was then converted to a 50% germination rate by taking the reciprocal of time (1/$x$). The 50% germination rate data was then plotted as a function of temperature for each field pea variety in SigmaPlot.

These plots were used to determine variety-specific cardinal temperatures (T_b_, T_o_, and T_m_) using a broken stick or triangular-shaped modelling approach, similar to the methods described by Olivier and Annandale^24^ and Andrade et al.^26^. The data for each pea variety was visually inspected for a linear trend of germination rate increase from the lowest-tested temperatures upward toward the presumed optimal temperature. Similarly, the data was visually inspected for a linear trend of germination rate decrease from near the presumed optimal temperature downward toward to the highest tested temperatures. If data points near the presumed optimal temperature deviated from the linear trends (i.e., germination rate plateaued over a temperature range), those points were not considered in analysis. SigmaPlot was used to run linear regression analysis (y = mx + b) separately over the datapoints below and above the optimal temperature. Cardinal temperatures were calculated using the resulting regression equations. The T_b_ was calculated by setting the germination rate equal to zero in the upward trending equation and solving for temperature. The T_m_ was calculated by doing the same thing using the downward trending equation. The T_o_ was calculated by setting the two equations equal to each other, then solving for temperature.

Analysis and statistical comparison of cardinal temperatures by seasonal classification (winter peas versus spring peas) was accomplished by normalizing the germination rate data to the variety-specific germination rate at T_o_. The normalized data was pooled by food-grade pea class and also across all food peas, keeping Granger AWP separate, then subjected to the same broken stick linear regression modeling approach described above using SigmaPlot. The software reported the root mean square error (RMSE) of the linear relationships. The individual RMSE values of the upward and downward germination rate by temperature response functions were used to determine the variance in T_b_ and T_m_, respectively, by dividing by the slope. The variance in T_o_ was determined by calculating a combined RMSE of paired upward and downward regression functions, modifying the functions by separately adding and subtracting the RMSE from the intercept (b) term, then calculating the maximum variance in temperature. This was done by setting select error-modified functions—upward + RMSE and downward − RMSE; downward + RMSE and upward − RMSE—equations equal to each other, solving for temperature, taking the difference between results, then dividing by two. A lack of overlap in RMSE between any two treatments was used to signify statistical differences.

Thermal time (τ) requirements to reach 50% germination were calculated using the approach used for pea previously by Olivier and Annandale^24^. The following criteria and equations were applied:

$$\tau \left( {^{^\circ } {\text{Cd}}} \right) = 0,\,\,\,\,{\text{when T}}_{{\text{b}}} > {\text{T}} > {\text{T}}_{{\text{m}}}$$

$$\tau \left( {^{^\circ } {\text{Cd}}} \right) = \left( {{\text{T}} - {\text{T}}_{{\text{b}}} } \right)\Delta {\text{t}},\,\,\,\,{\text{when T}}_{{\text{b}}} < {\text{T}} < {\text{T}}_{{\text{o}}}$$

$$\tau \left( {^\circ {\text{Cd}}} \right) = \left[ {\frac{{\left( {{\text{Tm}} - {\text{T}}} \right)\left( {{\text{To}} - {\text{Tb}}} \right)}}{{\left( {{\text{Tm}} - {\text{To}}} \right)}}} \right]\Delta t,\,\,\,\,{\text{when T}}_{{\text{b}}} < {\text{T}} < {\text{T}}_{{\text{o}}}$$

where, T is the average of daily minimum and maximum temperatures (°C), Δt is the time increment in days (d), and the cardinal temperatures (°C) are defined as above. As indicated, when T is below T_b_ or above T_m_, no thermal is accumulated. The first equation is used when T is between T_b_ and T_o_, indicating a linear increase in thermal time. The second equation was used when T is between T_o_ and T_m_, indicating a linear decrease in thermal time. Variety-specific estimates of cardinal temperatures and time to 50% germination were used to calculate thermal time at each tested temperature. Thermal time estimates were then averaged across temperatures for each variety and standard deviations were calculated. Averages and standard deviations were also calculated across all temperatures collectively for all winter food peas, spring food peas, and across all winter and spring.

The data collected on the pea seedlings at 10 days was analyzed using ANOVA in SAS with the MIXED procedure. The primary objective of this analysis was to understand the impacts of ambient temperature and seasonal classification of the peas (i.e., spring vs. winter peas) on growth traits of food peas. Granger AWP was excluded from the analysis, as it is not a food-grade pea and often had markedly different growth traits than all other varieties. Therefore, the fixed effects in the statistical model were temperature, season, and their interaction. Replication, pea variety, and their interaction were random effects in the model. The data were checked to ensure they satisfied the assumptions of normality and equal variances. This was done using Q-Q Plots, histograms, and plots of residuals. Due to violation of the assumptions, each response variable required transformation. The percent germination data was transformed by converting values to a fraction (dividing by 100), then applying the arcsine function. The shoot length and root length datasets were transformed by adding two to each value, then applying the log function. The root diameter data was transformed by directly applying the log function. Pairwise mean comparisons were made using Tukey’s method. Treatment effects were considered significant based on a threshold of *P* < 0.05.

In the below-freezing germination test, cumulative germination was determined on the days that observations were made. These values were averaged across replicate boxes for presentation.

## Electronic supplementary material

Below is the link to the electronic supplementary material.

Supplementary Material 1

## Data Availability

The data that support the findings of this study is presented within the manuscript and the raw dataset can be made available from the corresponding author upon request.

## Abbreviations

AWP: Austrian winter pea
RMSE: Root mean square error
SD: Standard deviation
T_b_: Base temperature
T_m_: Maximum temperature
T_o_: Optimal temperature
