# Supplementary material for: Field pea cardinal temperatures, growth and vigor traits of winter- and spring-adapted germplasm at germination and seedling stages
Source: Sci Rep. 2025 Jul 1;15:21759. doi: 10.1038/s41598-025-06342-w (PMC12218029; doi:10.1038/s41598-025-06342-w)
Supplement: Supplementary file 1 — Supplementary Material 1 [file 41598_2025_6342_MOESM1_ESM.pdf]

SUPPLEMENTAL MATERIAL

Table S1: Time and rate to 50% germination for all pea variety by temperature treatments included in this study.

Temp. (˚C)	Time (hr.)	Rate (per hr.)	Time (hr.)	Rate (per hr.)	Time (hr.)	Rate (per hr.)	Time (hr.)	Rate (per hr.)
	Granger AWP		*Vail*		*PS17100008*		*Dint*	
2	239	0.0042	254	0.0039	-[†]	-	345	0.0029
6	116	0.0086	129	0.0077	234	0.0043	164	0.0061
10	72.6	0.0138	83	0.0120	138	0.0072	118	0.0084
14	41.5	0.0241	74.3	0.0135	128	0.0078	68.8	0.0145
18	37.3	0.0268	52.5	0.0190	84.7	0.0118	58.3	0.0172
22	32.0	0.0313	45.1	0.0222	67.5	0.0148	52.6	0.0190
26	26.2	0.0382	47.1	0.0212	74.8	0.0134	47.6	0.0210
30	48.4	0.0207	51.3	0.0195	80.2	0.0125	50.8	0.0197
34	201	0.0050	152	0.0066	153	0.0065	133	0.0075
36	250	0.0040	197	0.0051	262	0.0038	-	-
	Kurtwood		*Passion*		*MiCa*		*Hampton*	
2	327	0.0031	348	0.0029	416	0.0024	315	0.0032
6	155	0.0064	151	0.0066	157	0.0064	178	0.0056
10	107	0.0094	112	0.0089	113	0.0089	113	0.0088
14	91.3	0.0110	97.1	0.0103	79.9	0.0125	101	0.0099
18	53.3	0.0188	54.9	0.0182	56.4	0.0177	68.7	0.0146
22	46.5	0.0215	54.1	0.0185	48.4	0.0207	54.8	0.0182
26	49	0.0204	57	0.0175	36	0.0278	58.1	0.0172
30	47.2	0.0212	50.8	0.0197	49	0.0204	57.6	0.0174
34	127	0.0079	102	0.0098	131	0.0076	93.8	0.0107
36	227	0.0044	207	0.0048	315	0.0032	253	0.0040
	Pro 171-7665		*Klondike*		*PS17100022*			
2	301	0.0033	431	0.0023	299	0.0033		
6	172	0.0058	182	0.0055	187	0.0053		
10	105	0.0095	131	0.0076	111	0.0090		
14	98.1	0.0102	90.7	0.0110	105	0.0095		
18	63	0.0159	65.9	0.0152	72.9	0.0137		
22	48	0.0208	57.1	0.0175	54.6	0.0183		
26	59.7	0.0168	57.3	0.0175	58.8	0.0170		
30	53.6	0.0187	52.7	0.0190	65.3	0.0153		
34	151.7	0.0066	-	-	167	0.0060		
36	210.5	0.0048	213	0.0047	242	0.0041		

[†]Missing values signify that 50% germination was not achieved.
